# *trans*-Tetra­aqua­bis­(isonicotinamide-κ*N* ^1^)cobalt(II) bis­(3-hy­droxy­benzoate) tetra­hydrate

**DOI:** 10.1107/S1600536812003911

**Published:** 2012-02-04

**Authors:** İIbrahim Göker Zaman, Nagihan Çaylak Delibaş, Hacali Necefoğlu, Tuncer Hökelek

**Affiliations:** a Department of Chemistry, Kafkas University, 36100 Kars, Turkey; b Department of Physics, Sakarya University, 54187 Esentepe, Sakarya, Turkey; c Department of Physics, Hacettepe University, 06800 Beytepe, Ankara, Turkey

## Abstract

The asymmetric unit of the title compound, [Co(C_6_H_6_N_2_O)_2_(H_2_O)_4_](C_7_H_5_O_3_)_2_·4H_2_O, contains one-half of the complex cation with the Co^II^ ion located on an inversion center, a 3-hy­droxy­benzoate counter-anion and two uncoordinated water mol­ecules. Four water O atoms in the equatorial plane around the Co^II^ ion [Co—O = 2.0593 (16) and 2.1118 (16) Å] form a slightly distorted square-planar arrangement, and the distorted octahedral geometry is completed by the two N atoms [Co—N = 2.1306 (18) Å] from two isonicotinamide ligands. In the anion, the carboxyl­ate group is twisted from the attached benzene ring at 8.84 (17)°. In the crystal, a three-dimensional hydrogen-bonding network, formed by classical O—H⋯O and N—H⋯O hydrogen bonds, consolidates the crystal packing, which exhibits π–π inter­actions between the benzene and pyridine rings, with centroid–centroid distances of 3.458 (1) and 3.606 (1) Å, respectively.

## Related literature

For related structures, see: Hökelek, Dal, Tercan, Özbek *et al.* (2009 ▶); Hökelek, Dal, Tercan, Aybirdi *et al.* (2009 ▶); Hökelek, Yılmaz, Tercan, Gürgen *et al.* (2009 ▶); Hökelek, Yılmaz, Tercan, Özbek *et al.* (2009 ▶); Hökelek, Yılmaz, Tercan, Sertçelik *et al.* (2009 ▶); Sertçelik *et al.* (2009**a* ▶,b* ▶); Zaman *et al.* (2012 ▶).
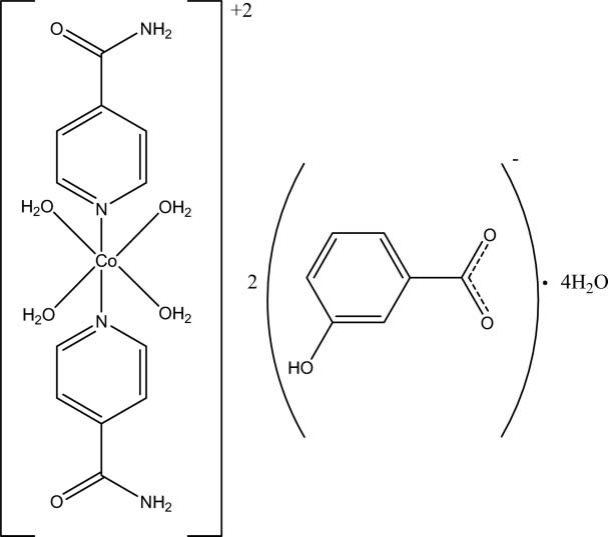


## Experimental

### 

#### Crystal data

[Co(C_6_H_6_N_2_O)_2_(H_2_O)_4_](C_7_H_5_O_3_)_2_·4H_2_O
*M* *_r_* = 721.53
Monoclinic, 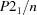

*a* = 6.7032 (2) Å
*b* = 17.0523 (4) Å
*c* = 13.5406 (3) Å
β = 100.194 (3)°
*V* = 1523.32 (7) Å^3^
*Z* = 2
Mo *K*α radiation
μ = 0.65 mm^−1^
*T* = 100 K
0.29 × 0.28 × 0.14 mm

#### Data collection

Bruker Kappa APEXII CCD area-detector diffractometer
Absorption correction: multi-scan (*SADABS*; Bruker, 2005 ▶) *T*_min_ = 0.840, *T*_max_ = 0.916
14243 measured reflections
3779 independent reflections
3590 reflections with *I* > 2σ(*I*)
*R*_int_ = 0.019

#### Refinement

*R*[*F* ^2^ > 2σ(*F* ^2^)] = 0.034
*wR*(*F* ^2^) = 0.086
*S* = 1.23
3779 reflections
258 parameters
16 restraints
H atoms treated by a mixture of independent and constrained refinement
Δρ_max_ = 0.47 e Å^−3^
Δρ_min_ = −0.42 e Å^−3^

### 

Data collection: *APEX2* (Bruker, 2007 ▶); cell refinement: *SAINT* (Bruker, 2007 ▶); data reduction: *SAINT*; program(s) used to solve structure: *SHELXS97* (Sheldrick, 2008 ▶); program(s) used to refine structure: *SHELXL97* (Sheldrick, 2008 ▶); molecular graphics: *Mercury* (Macrae *et al.*, 2006 ▶); software used to prepare material for publication: *WinGX* (Farrugia, 1999 ▶) and *PLATON* (Spek, 2009 ▶).

## Supplementary Material

Crystal structure: contains datablock(s) I, global. DOI: 10.1107/S1600536812003911/cv5239sup1.cif

Structure factors: contains datablock(s) I. DOI: 10.1107/S1600536812003911/cv5239Isup2.hkl

Additional supplementary materials:  crystallographic information; 3D view; checkCIF report

## Figures and Tables

**Table 1:** Hydrogen-bond geometry (Å, °)

*D*—H⋯*A*	*D*—H	H⋯*A*	*D*⋯*A*	*D*—H⋯*A*
N2—H21⋯O2^i^	0.86 (3)	2.18 (3)	3.031 (3)	170 (3)
N2—H22⋯O8^ii^	0.85 (4)	2.20 (4)	3.007 (3)	158 (3)
O3—H31⋯O7	0.91 (4)	1.81 (4)	2.711 (2)	170 (4)
O5—H51⋯O3^ii^	0.96 (3)	1.76 (3)	2.715 (2)	171 (3)
O5—H52⋯O2^iii^	0.86 (3)	1.95 (4)	2.782 (2)	163 (4)
O6—H61⋯O4^iv^	0.95 (3)	1.73 (3)	2.685 (2)	178 (4)
O6—H62⋯O2^v^	0.82 (3)	1.89 (4)	2.683 (2)	161 (3)
O7—H71⋯O1^i^	0.98 (3)	1.76 (4)	2.740 (3)	179 (3)
O8—H81⋯O1	0.96 (4)	1.81 (4)	2.760 (3)	174 (3)
O8—H82⋯O7^vi^	0.83 (5)	2.06 (4)	2.807 (3)	149 (5)

Symmetry codes: (i) 
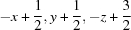
; (ii) 
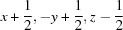
; (iii) 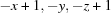; (iv) 
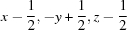
; (v) 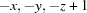; (vi) 
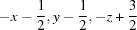
.

**Fig. 1.**
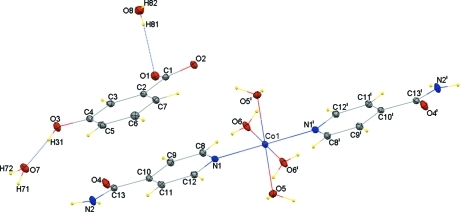
The molecular structure of (I) with the atom-numbering scheme [symmetry code: (') -x, -y, -z]. Displacement ellipsoids are drawn at the 50% probability level. Hydrogen bonds are shown as dashed lines.

## supplementary crystallographic information

### Comment

As a part of our ongoing investigation on transition metal complexes of nicotinamide (NA) and/or the nicotinic acid derivative *N*,*N*-diethylnicotinamide (DENA) (Hökelek, Dal, Tercan, Özbek *et al.*, 2009; Hökelek, Dal, Tercan, Aybirdi *et al.*, 2009; Hökelek, Yılmaz, Tercan, Gürgen *et al.*, 2009; Hökelek, Yılmaz, Tercan, Özbek *et al.*, 2009; Hökelek, Yılmaz, Tercan, Sertçelik *et al.*, 2009; Sertçelik *et al.*, 2009**a*,b*), the title compound was synthesized and its crystal structure is reported herein.

The title compound (I) is isostructural with the related Ni complex (Zaman *et al.*, 2012). In (I) (Fig. 1), four O atoms (O5, O6, and the symmetry-related atoms, O5', O6') in the equatorial plane around the Co atom form a slightly distorted square-planar arrangement, while the slightly distorted octahedral coordination is completed by the two pyridine N atoms (N1, N1') of the INA ligands at 2.1306 (18) Å from the Co atom in the axial positions (Fig. 1). The average Co—O bond length is 2.0856 (16) Å. The intramolecular O—H···O hydrogen bonds (Table 1) link the uncoordinated water molecules to the HB anion. The dihedral angle between the planar carboxylate group (O1/O2/C1) and the benzene ring A (C2—C7) is 8.84 (17)°, while that between rings A and B (N1/C8—C12) is 1.24 (7)°.

In the crystal structure, intermolecular O—H···O and N—H···O hydrogen bonds (Table 1) link the molecules into a three-dimensional network, in which they may be effective in the stabilization of the structure. π–π Contacts between the benzene and pyridine rings, *Cg*1—*Cg*2 and *Cg*1—*Cg*2^i^, [symmetry code: (i) -1 + *x*, *y*, *z*, where *Cg*1 and *Cg*2 are centroids of the rings A (C2—C7) and B (N1/C8—C12), respectively] may further stabilize the structure, with centroid-centroid distances of 3.606 (1) and 3.458 (1) Å, respectively.

### Experimental

The title compound was prepared by the reaction of CoSO_4_.7H_2_O (1.406 g, 5 mmol) in H_2_O (100 ml) and INA (1.220 g, 10 mmol) in H_2_O (50 ml) with sodium 3-hydroxybenzoate (1.601 g, 10 mmol) in H_2_O (100 ml). The mixture was filtered and set aside to crystallize at ambient temperature for three weeks, giving orange single crystals.

### Refinement

Atoms H51, H52, H61, H62, H71, H72, H81 and H82 (for H_2_O), H21 and H22 (for NH_2_) and H31 (for OH) were located in difference Fourier map and were refined by applying bond length restraints. The C-bound H-atoms were positioned geometrically (C—H = 0.93 Å) and constrained to ride on their parent atoms, with *U*_iso_(H) = 1.2 × *U*_eq_(C).

### Crystal data

**Table:**

[Co(C_6_H_6_N_2_O)_2_(H_2_O)_4_](C_7_H_5_O_3_)_2_·4H_2_O	*F*(000) = 754
*M**_r_* = 721.53	*D*_x_ = 1.573 Mg m^−^^3^
Monoclinic, *P*2_1_/*n*	Mo *K*α radiation, λ = 0.71073 Å
Hall symbol: -P 2yn	Cell parameters from 9057 reflections
*a* = 6.7032 (2) Å	θ = 2.4–28.4°
*b* = 17.0523 (4) Å	µ = 0.65 mm^−^^1^
*c* = 13.5406 (3) Å	*T* = 100 K
β = 100.194 (3)°	Block, orange
*V* = 1523.32 (7) Å^3^	0.29 × 0.28 × 0.14 mm
*Z* = 2	

### Data collection

**Table:**

Bruker Kappa APEXII CCD area-detector diffractometer	3779 independent reflections
Radiation source: fine-focus sealed tube	3590 reflections with *I* > 2σ(*I*)
Graphite monochromator	*R*_int_ = 0.019
φ and ω scans	θ_max_ = 28.4°, θ_min_ = 2.4°
Absorption correction: multi-scan (*SADABS*; Bruker, 2005)	*h* = −8→8
*T*_min_ = 0.840, *T*_max_ = 0.916	*k* = −22→21
14243 measured reflections	*l* = −15→18

### Refinement

**Table:**

Refinement on *F*^2^	Primary atom site location: structure-invariant direct methods
Least-squares matrix: full	Secondary atom site location: difference Fourier map
*R*[*F*^2^ > 2σ(*F*^2^)] = 0.034	Hydrogen site location: inferred from neighbouring sites
*wR*(*F*^2^) = 0.086	H atoms treated by a mixture of independent and constrained refinement
*S* = 1.23	*w* = 1/[σ^2^(*F*_o_^2^) + (0.0269*P*)^2^ + 1.4849*P*] where *P* = (*F*_o_^2^ + 2*F*_c_^2^)/3
3779 reflections	(Δ/σ)_max_ < 0.001
258 parameters	Δρ_max_ = 0.47 e Å^−^^3^
16 restraints	Δρ_min_ = −0.42 e Å^−^^3^

### Special details

**Table:**

Geometry. All e.s.d.'s (except the e.s.d. in the dihedral angle between two l.s. planes) are estimated using the full covariance matrix. The cell e.s.d.'s are taken into account individually in the estimation of e.s.d.'s in distances, angles and torsion angles; correlations between e.s.d.'s in cell parameters are only used when they are defined by crystal symmetry. An approximate (isotropic) treatment of cell e.s.d.'s is used for estimating e.s.d.'s involving l.s. planes.
Refinement. Refinement of *F*^2^ against ALL reflections. The weighted *R*-factor *wR* and goodness of fit *S* are based on *F*^2^, conventional *R*-factors *R* are based on *F*, with *F* set to zero for negative *F*^2^. The threshold expression of *F*^2^ > σ(*F*^2^) is used only for calculating *R*-factors(gt) *etc*. and is not relevant to the choice of reflections for refinement. *R*-factors based on *F*^2^ are statistically about twice as large as those based on *F*, and *R*- factors based on ALL data will be even larger.

### Fractional atomic coordinates and isotropic or equivalent isotropic displacement parameters (Å^2^)

**Table:**

	*x*	*y*	*z*	*U*_iso_*/*U*_eq_	
Co1	0.5000	0.0000	0.5000	0.00895 (12)	
O1	0.1791 (2)	0.11648 (9)	0.83771 (12)	0.0148 (3)	
O2	0.0778 (2)	0.04189 (9)	0.70323 (12)	0.0136 (3)	
O3	0.0160 (3)	0.39111 (10)	0.72117 (13)	0.0160 (3)	
H31	0.005 (6)	0.430 (2)	0.675 (3)	0.045 (11)*	
O4	0.6007 (3)	0.33160 (9)	0.81945 (12)	0.0166 (3)	
O5	0.6814 (2)	0.04700 (10)	0.40186 (12)	0.0141 (3)	
H51	0.627 (6)	0.074 (2)	0.341 (2)	0.057 (12)*	
H52	0.759 (5)	0.0138 (18)	0.380 (3)	0.039 (10)*	
O6	0.2333 (2)	0.03327 (10)	0.40967 (13)	0.0157 (3)	
H61	0.190 (5)	0.0815 (12)	0.378 (3)	0.039 (10)*	
H62	0.151 (5)	0.0013 (15)	0.381 (3)	0.039 (10)*	
O7	−0.0014 (3)	0.51872 (10)	0.60055 (13)	0.0168 (3)	
H71	0.113 (5)	0.554 (2)	0.623 (3)	0.056 (12)*	
H72	−0.006 (10)	0.516 (4)	0.541 (2)	0.13 (3)*	
O8	−0.0938 (3)	0.06685 (11)	0.95512 (13)	0.0195 (4)	
H81	−0.006 (5)	0.086 (2)	0.912 (3)	0.049 (11)*	
H82	−0.200 (6)	0.054 (4)	0.917 (4)	0.16 (3)*	
N1	0.5124 (3)	0.11063 (11)	0.57398 (14)	0.0107 (3)	
N2	0.4839 (3)	0.39703 (12)	0.67598 (15)	0.0144 (4)	
H21	0.473 (5)	0.441 (2)	0.705 (2)	0.023 (8)*	
H22	0.440 (5)	0.396 (2)	0.613 (3)	0.030 (9)*	
C1	0.1091 (3)	0.10798 (13)	0.74547 (16)	0.0112 (4)	
C2	0.0580 (3)	0.18067 (12)	0.68279 (16)	0.0106 (4)	
C3	0.0646 (3)	0.25369 (13)	0.72979 (16)	0.0118 (4)	
H3	0.1019	0.2575	0.7991	0.014*	
C4	0.0151 (3)	0.32060 (13)	0.67240 (17)	0.0122 (4)	
C5	−0.0363 (3)	0.31556 (13)	0.56836 (17)	0.0141 (4)	
H5	−0.0678	0.3607	0.5302	0.017*	
C6	−0.0403 (3)	0.24293 (14)	0.52186 (17)	0.0141 (4)	
H6	−0.0735	0.2395	0.4523	0.017*	
C7	0.0052 (3)	0.17518 (13)	0.57864 (17)	0.0126 (4)	
H7	0.0004	0.1265	0.5473	0.015*	
C8	0.5612 (3)	0.11620 (13)	0.67453 (16)	0.0117 (4)	
H8	0.5880	0.0703	0.7117	0.014*	
C9	0.5732 (3)	0.18675 (13)	0.72507 (17)	0.0124 (4)	
H9	0.6091	0.1881	0.7946	0.015*	
C10	0.5308 (3)	0.25590 (12)	0.67073 (16)	0.0108 (4)	
C11	0.4790 (3)	0.25056 (13)	0.56688 (17)	0.0122 (4)	
H11	0.4491	0.2955	0.5281	0.015*	
C12	0.4724 (3)	0.17733 (13)	0.52206 (16)	0.0119 (4)	
H12	0.4386	0.1744	0.4525	0.014*	
C13	0.5415 (3)	0.33216 (13)	0.72758 (17)	0.0118 (4)	

### Atomic displacement parameters (Å^2^)

**Table:**

	*U*^11^	*U*^22^	*U*^33^	*U*^12^	*U*^13^	*U*^23^
Co1	0.01097 (19)	0.0073 (2)	0.00830 (19)	0.00020 (14)	0.00093 (14)	−0.00006 (14)
O1	0.0186 (8)	0.0140 (8)	0.0116 (7)	0.0004 (6)	0.0015 (6)	0.0008 (6)
O2	0.0155 (7)	0.0089 (7)	0.0159 (8)	−0.0015 (6)	0.0015 (6)	−0.0008 (6)
O3	0.0239 (8)	0.0094 (7)	0.0144 (8)	−0.0002 (6)	0.0022 (6)	−0.0015 (6)
O4	0.0260 (9)	0.0117 (8)	0.0113 (8)	−0.0024 (6)	0.0011 (6)	−0.0020 (6)
O5	0.0170 (8)	0.0133 (8)	0.0132 (7)	0.0007 (6)	0.0060 (6)	0.0012 (6)
O6	0.0159 (8)	0.0094 (7)	0.0188 (8)	0.0001 (6)	−0.0048 (6)	0.0009 (6)
O7	0.0196 (8)	0.0134 (8)	0.0164 (8)	−0.0028 (6)	0.0006 (7)	0.0005 (6)
O8	0.0235 (9)	0.0188 (9)	0.0177 (8)	−0.0023 (7)	0.0076 (7)	−0.0004 (7)
N1	0.0102 (8)	0.0107 (8)	0.0113 (8)	−0.0007 (6)	0.0020 (6)	−0.0010 (7)
N2	0.0211 (9)	0.0097 (9)	0.0117 (9)	0.0005 (7)	0.0014 (8)	−0.0025 (7)
C1	0.0091 (9)	0.0118 (10)	0.0134 (10)	−0.0006 (7)	0.0035 (7)	0.0011 (8)
C2	0.0094 (9)	0.0104 (10)	0.0125 (10)	−0.0010 (7)	0.0027 (7)	0.0016 (7)
C3	0.0119 (9)	0.0127 (10)	0.0108 (9)	−0.0021 (7)	0.0016 (8)	−0.0003 (8)
C4	0.0110 (9)	0.0098 (10)	0.0159 (10)	−0.0008 (7)	0.0023 (8)	−0.0016 (8)
C5	0.0145 (10)	0.0121 (10)	0.0153 (10)	−0.0012 (8)	0.0018 (8)	0.0033 (8)
C6	0.0145 (10)	0.0163 (11)	0.0110 (10)	−0.0013 (8)	0.0009 (8)	0.0001 (8)
C7	0.0125 (9)	0.0116 (10)	0.0139 (10)	−0.0021 (7)	0.0028 (8)	−0.0017 (8)
C8	0.0132 (9)	0.0095 (9)	0.0122 (10)	0.0000 (7)	0.0023 (8)	0.0012 (8)
C9	0.0127 (9)	0.0128 (10)	0.0116 (10)	−0.0006 (8)	0.0014 (8)	−0.0003 (8)
C10	0.0094 (9)	0.0092 (9)	0.0138 (10)	−0.0011 (7)	0.0025 (7)	−0.0013 (8)
C11	0.0136 (9)	0.0094 (10)	0.0132 (10)	−0.0008 (7)	0.0019 (8)	0.0008 (8)
C12	0.0129 (9)	0.0115 (10)	0.0114 (10)	−0.0012 (7)	0.0022 (8)	−0.0007 (8)
C13	0.0120 (9)	0.0108 (10)	0.0132 (10)	−0.0026 (7)	0.0039 (8)	−0.0019 (8)

### Geometric parameters (Å, º)

**Table:**

Co1—O5	2.1118 (16)	N2—H21	0.85 (3)
Co1—O5^i^	2.1118 (16)	N2—H22	0.85 (4)
Co1—O6	2.0593 (16)	C2—C1	1.507 (3)
Co1—O6^i^	2.0593 (16)	C2—C3	1.396 (3)
Co1—N1	2.1306 (18)	C2—C7	1.395 (3)
Co1—N1^i^	2.1306 (18)	C3—H3	0.9300
O1—C1	1.262 (3)	C4—C3	1.387 (3)
O2—C1	1.264 (3)	C5—C4	1.392 (3)
O3—C4	1.371 (3)	C5—C6	1.388 (3)
O3—H31	0.91 (4)	C5—H5	0.9300
O4—C13	1.237 (3)	C6—H6	0.9300
O5—H51	0.963 (18)	C7—C6	1.391 (3)
O5—H52	0.85 (2)	C7—H7	0.9300
O6—H61	0.948 (17)	C8—C9	1.379 (3)
O6—H62	0.82 (2)	C8—H8	0.9300
O7—H71	0.978 (18)	C9—C10	1.392 (3)
O7—H72	0.81 (2)	C9—H9	0.9300
O8—H81	0.960 (18)	C11—C10	1.390 (3)
O8—H82	0.83 (3)	C11—C12	1.386 (3)
N1—C8	1.346 (3)	C11—H11	0.9300
N1—C12	1.339 (3)	C12—H12	0.9300
N2—C13	1.328 (3)	C13—C10	1.506 (3)
			
O5^i^—Co1—O5	180.0	C7—C2—C3	120.2 (2)
O5—Co1—N1	88.91 (7)	C2—C3—H3	120.2
O5—Co1—N1^i^	91.09 (7)	C4—C3—C2	119.6 (2)
O5^i^—Co1—N1	91.09 (7)	C4—C3—H3	120.2
O5^i^—Co1—N1^i^	88.91 (7)	O3—C4—C3	118.1 (2)
O6—Co1—O5	93.32 (7)	O3—C4—C5	121.4 (2)
O6—Co1—O5^i^	86.68 (7)	C3—C4—C5	120.5 (2)
O6^i^—Co1—O5^i^	93.32 (7)	C4—C5—H5	120.1
O6^i^—Co1—O5	86.68 (7)	C6—C5—C4	119.7 (2)
O6—Co1—O6^i^	180.0	C6—C5—H5	120.1
O6—Co1—N1	89.59 (7)	C5—C6—C7	120.4 (2)
O6^i^—Co1—N1	90.41 (7)	C5—C6—H6	119.8
O6—Co1—N1^i^	90.41 (7)	C7—C6—H6	119.8
O6^i^—Co1—N1^i^	89.59 (7)	C2—C7—H7	120.2
N1^i^—Co1—N1	180.0	C6—C7—C2	119.6 (2)
C4—O3—H31	108 (2)	C6—C7—H7	120.2
Co1—O5—H51	123 (2)	N1—C8—C9	123.1 (2)
Co1—O5—H52	115 (2)	N1—C8—H8	118.5
H52—O5—H51	101 (3)	C9—C8—H8	118.5
Co1—O6—H61	132 (2)	C8—C9—C10	119.2 (2)
Co1—O6—H62	123 (2)	C8—C9—H9	120.4
H62—O6—H61	104 (2)	C10—C9—H9	120.4
H71—O7—H72	104 (3)	C9—C10—C13	118.31 (19)
H81—O8—H82	105 (3)	C11—C10—C9	118.0 (2)
C8—N1—Co1	121.36 (14)	C11—C10—C13	123.65 (19)
C12—N1—Co1	121.18 (14)	C10—C11—H11	120.5
C12—N1—C8	117.46 (19)	C12—C11—C10	119.0 (2)
C13—N2—H21	122 (2)	C12—C11—H11	120.5
C13—N2—H22	121 (2)	N1—C12—C11	123.2 (2)
H21—N2—H22	116 (3)	N1—C12—H12	118.4
O1—C1—O2	123.5 (2)	C11—C12—H12	118.4
O1—C1—C2	118.07 (19)	O4—C13—N2	123.2 (2)
O2—C1—C2	118.38 (19)	O4—C13—C10	119.00 (19)
C3—C2—C1	119.43 (19)	N2—C13—C10	117.83 (19)
C7—C2—C1	120.39 (19)		
			
O5—Co1—N1—C8	−130.62 (16)	C1—C2—C7—C6	−179.56 (19)
O5^i^—Co1—N1—C8	49.38 (16)	C3—C2—C7—C6	0.2 (3)
O5—Co1—N1—C12	49.21 (16)	O3—C4—C3—C2	177.66 (19)
O5^i^—Co1—N1—C12	−130.79 (16)	C5—C4—C3—C2	−1.5 (3)
O6—Co1—N1—C8	136.06 (16)	C6—C5—C4—O3	−178.4 (2)
O6^i^—Co1—N1—C8	−43.94 (16)	C6—C5—C4—C3	0.7 (3)
O6—Co1—N1—C12	−44.12 (16)	C4—C5—C6—C7	0.5 (3)
O6^i^—Co1—N1—C12	135.88 (16)	C2—C7—C6—C5	−1.0 (3)
Co1—N1—C8—C9	179.20 (16)	N1—C8—C9—C10	0.8 (3)
C12—N1—C8—C9	−0.6 (3)	C8—C9—C10—C11	−0.4 (3)
Co1—N1—C12—C11	−179.88 (16)	C8—C9—C10—C13	178.98 (19)
C8—N1—C12—C11	0.0 (3)	C12—C11—C10—C9	−0.3 (3)
C3—C2—C1—O1	−8.0 (3)	C12—C11—C10—C13	−179.57 (19)
C3—C2—C1—O2	171.26 (19)	C10—C11—C12—N1	0.5 (3)
C7—C2—C1—O1	171.73 (19)	O4—C13—C10—C9	5.0 (3)
C7—C2—C1—O2	−9.0 (3)	O4—C13—C10—C11	−175.7 (2)
C1—C2—C3—C4	−179.20 (19)	N2—C13—C10—C9	−174.3 (2)
C7—C2—C3—C4	1.1 (3)	N2—C13—C10—C11	5.0 (3)

Symmetry code: (i) −*x*+1, −*y*, −*z*+1.

### Hydrogen-bond geometry (Å, º)

**Table:**

*D*—H···*A*	*D*—H	H···*A*	*D*···*A*	*D*—H···*A*
N2—H21···O2^ii^	0.86 (3)	2.18 (3)	3.031 (3)	170 (3)
N2—H22···O8^iii^	0.85 (4)	2.20 (4)	3.007 (3)	158 (3)
O3—H31···O7	0.91 (4)	1.81 (4)	2.711 (2)	170 (4)
O5—H51···O3^iii^	0.96 (3)	1.76 (3)	2.715 (2)	171 (3)
O5—H52···O2^i^	0.86 (3)	1.95 (4)	2.782 (2)	163 (4)
O6—H61···O4^iv^	0.95 (3)	1.73 (3)	2.685 (2)	178 (4)
O6—H62···O2^v^	0.82 (3)	1.89 (4)	2.683 (2)	161 (3)
O7—H71···O1^ii^	0.98 (3)	1.76 (4)	2.740 (3)	179 (3)
O8—H81···O1	0.96 (4)	1.81 (4)	2.760 (3)	174 (3)
O8—H82···O7^vi^	0.83 (5)	2.06 (4)	2.807 (3)	149 (5)

Symmetry codes: (i) −*x*+1, −*y*, −*z*+1; (ii) −*x*+1/2, *y*+1/2, −*z*+3/2; (iii) *x*+1/2, −*y*+1/2, *z*−1/2; (iv) *x*−1/2, −*y*+1/2, *z*−1/2; (v) −*x*, −*y*, −*z*+1; (vi) −*x*−1/2, *y*−1/2, −*z*+3/2.
